# Notes and News

**Published:** 1891-01-24

**Authors:** 


### Notes and News.

Collected and Communicated.

A grand fête in aid of the West-end Hospital and School of Massage, Welbeck-street, is announced for the first week in March, and is to take place at the Royal Albert Hall. Attempts will be made to realise the antediluvian costumes and dwellings, as well as the subterranean surroundings of our supposed future conquerors, as depicted in Lord Lytton's novel, "The Coming Race." The Duke of Portland (President of the Hospital) will take part as an honorary steward.

Sir Edwin Arnold gives an interesting account of the splendid way in which the Japanese officials deal with a visitation of cholera, such as they are now enduring. Their central idea is to isolate each case as it occurs, and, as the police are pretty well omnipotent, this is not so difficult as it would prove elsewhere. As soon as a case is declared, a policeman arrives at the house and carries off the patient to the hospital. The people dread this intensely; indeed, their nature is so sensitive that many women and even men die actually from the depressing fact of being there, in spite of good treatment, kindly and brave nursing, and fearless and devoted medical assistance. Fear of going there makes the poor people conceal the beginning of the attack, and allow the insidious symptoms to go on, hoping to pull through, thus giving medical science too little opportunity of action and causing the malady to be fatal in seventy cases out of a hundred. The rigid system of isolation is nevertheless kept up, and to this is due the fact that the disease passes from individual to individual, without any great leaps and bounds, and that the daily returns are now happily declining. Moreover, the hot weather is coming to an end. Constant showers of rain have flushed all the open drains and ditches, so that if there is no recrudescence of the dreadful plague it may be hoped that Japan may be quit this year of the penalty of her neighbourhood to China, with a death tribute of not more than 20 000 lives. How long will science allow the cholera bacillus to carry off its thousands in this way? Sir Edwin Arnold speaks very highly of the bravery and good sense shown by the Japanese people. At no time has there been the slightest difficulty in procuring nurses, bearers, and people to disinfect and carry away the corpses.

The following vacancies are announced this week, the amount of salary in each case being given after the name of the institution:—

**Resident Medical Officers.**

Hospital for Infectious Diseases, Newcastle-upon-Tyne, Medical Assistant—£50, board, &c.
Central London Ophthalmic Hospital, House Surgeon-rooms.
City of London Lunatic Asylum, Dartford, Assistant Medical Officer—£150, board, &c.
Brixton, Streatham, and Herne Hill Dispensary, House Surgeon—£150, rooms.
Turner's Hospital, Kirkleatham, Surgeon—board, &c.
Royal Portsmouth Hospital, House Surgeon—£70, board, &c.
Royal Albert Hospital, Devonport, Assistant House Surgeon—board, &c.
General Hospital, Birmingham, Assistant House Surgeon—board, &c.
Lancashire County Asylum, Pathological Assistant Medical Officer—£200, board, &c.
Dundee Royal Lunatic Asylum, Assistant Medical Officer—£100, board, &c.
Dundee Royal Lunatic Asylum, Clinical Assistant—board, &c.

**Non-Resident Medical Officers.**

Charing Cross Hospital, Assistant Surgeons.
Metropolitan Hospital, Dental Surgeons.
Infirmary for Consumption and Diseases of the Chest and Throat, Visiting Physicians.
County Council of Ayrshire, County Medical Officer—£40.
County of Dumfries, Medical Officer—£300.
Parish of Tongue, Sutherlandshire, Medical Officer—£165, house.

Thanks to the munificent generosity of Mr. A. M. Singer and Mr. W. Singer, there will shortly be opened at Paignton, a Cottage Hospital and Provident Dispensary, for the benefit of the residents in the parishes of Paignton and Marldon. The above-named gentlemen presented the site, defrayed the cost of the handsome new buildings, and fitted and furnished the hospital throughout; and have, moreover, donated more than £2,000 to be invested as endowment. The interest on this sum, however, is insufficient to meet the cost of maintenance, and it is the desire of the donors that this shall be met chiefly by the subscriptions and donations of subscribers, to whom will be handed over the management of the institution. The hospital is constructed on the best modern principles, and is as handsome and imposing as it is convenient and commodious.

Of the many useful and benevolent medical charities that solicit assistance about this time of year, the Devonshire Hospital and Buxton Bath Charity may be classed among the most deserving. For centuries the charity has given relief to poor rheumatic sufferers. The curative value of the Buxton mineral waters, in cases of rheumatism and gout, has much, of course, to do with the signal benefits derived by such patients of the hospital, but praise is also due to the excellent medical and nursing staff, who have shown a skill and care in the treatment of the sufferers which ought not to go unrecognised. Some years ago the Devonshire Hospital was aided by a grant of £24,000 from the governors of the Cotton District Convalescent Fund. To Manchester and Lancashire generally, the hospital is of great importance, and its continued prosperity is a matter of no little concern to the people of that district.

The fifty-fifth annual report of the Committee of Management of the Princess Louise Home shows that 1890 has been a year of progress. More girls have been admitted, and improvements in their clothing and accommodation have been made. The girls have now for the first time been provided with a mackintosh and ulster each, as well as other additional articles of clothing. Four bathing cubicles have been erected, fitted with full sized baths of iron and hot and cold water services, which form a great addition to the institution. The receipts for the year amounted to £1,059 9s., and the expenditure was £1.933 3s. 7d., leaving a balance on the right side of £126 5s. 5d. Since the establishment of the Home 1,536 girls have been admitted, the majority of these are sent out to service. It is curious to note that out of this large number only one girl has emigrated.

